# My journey to ASCI: why ASCI is so important to me

**DOI:** 10.1172/JCI163625

**Published:** 2022-12-15

**Authors:** Hossein Ardehali

It has been an immense privilege to serve as the President of ASCI this past year and to serve on the Council since 2016. I will conclude my term with a sense of honor knowing that, together, we accomplished a number of important initiatives during my tenure. I also feel privileged to have had the opportunity to work with a group of exceptional scientists on the Council and a highly dedicated and superb ASCI administrative staff.

ASCI represents two elements that I cherish dearly in my life: science and medicine. In this presidential address, I will detail how my heritage and early life experiences got me on the path of being a physician-scientist. I recognize that I likely come from a background that very few previous ASCI presidents have experienced, as I was not born in this country and I spent a major part of my childhood growing up in a war-torn country. My experience growing up in a country that witnessed a disruptive revolution and a bloody war will always remain with me and continues to influence many aspects of my life.

I was born in a large middle-class family in Iran. My father was an economist who was educated in the US. His family was almost exclusively in the business of selling Persian rugs and being landlords. He was one of the first to go to college, and after he finished his undergraduate studies, he decided to immigrate to the US for his graduate studies (Figure 1). There were no direct flights from Iran to the US in 1948, so he flew from Tehran to Beirut and then went on a ship from Beirut to New York, a journey that took him almost one month. When he arrived in the US, he was fascinated by the technology and advancement of this country. He told us several stories when we were children about his first few days in the US. One story in particular stayed with me over the years. The story begins with my father going for a walk in New York City after his arrival, and he was astonished that at every intersection, cars would stop all of the sudden without a policeman present. After inspecting the cars for several minutes, he could not figure out the reason and concluded that there must be a magnet in the ground that stopped the cars! It did not occur to him to look up and note that there is a light that changes colors to signal the traffic. As he continued his walk, he was later stopped by the police, asking him, “What are you doing walking on a highway?” His response was, of course, “What is a highway?”

He received his PhD in economics from Clark University in Massachusetts and returned to Iran in 1952. His experience in the US had a profound impact on him and shaped his way of thinking. He was a big admirer of American culture, technology, and science and was a firm believer that for Iran to get to the level of advanced countries, it needed to cooperate with the US extensively. This desire pushed him to work for the US-sponsored Point Four Program, created by US President Harry Truman in 1949 to provide financial and technical assistance to underdeveloped countries with the objective to prevent the expansion of communism. This program helped to keep Iran a close US ally and free of communism, showing how American foreign policy can work to mutually benefit the citizens of both nations.

I am the sixth child in a family of seven children. During my childhood, the most memorable topics discussed by my parents with us were focused on interpersonal and moral issues. Prominent topics included sensitivity to others’ feelings, altruism, honesty, and tolerance. For my father, ideals and integrity were of upmost importance, while my mother encouraged us to focus on our education. My oldest sister and my brothers immigrated to the United States during their high school years. I was five years old when my oldest sister moved to the US. I remember the night before her trip, and as a child, I kept thinking to myself how someone can leave everything behind to move to a new environment at such a young age, not realizing that this would one day happen to me (Figure 2).

My serene childhood was disrupted by two events: the Iranian Revolution when I was ten years old and the death of my sister when I was twelve. The path of progress towards an advanced country in Iran was disrupted by a mob that promoted violence and anti-Western propaganda. My family was among the first to oppose the Revolution, but they could not voice their opposition publicly, since the new government would sentence anyone who was in opposition to jail or possibly death. In the years following the Revolution, Iran and Iraq got into a bloody war that lasted eight years. At that time, the shadow of the new regime and the devastating war had made Iran a tense country. Through certain channels, my mother got my younger brother and myself out of Iran when I was 16 years old. We eventually joined the rest of my family in the United States five months after leaving Iran. My experience leaving Iran at a challenging time reinforced my desire and aspiration to excel in my educational endeavors.

After finishing high school in California and college at the University of Utah, majoring in computer science, I attended Vanderbilt, where I got my MD and PhD degrees as part of the Medical Scientist Training Program (MSTP). I completed my PhD in the lab of Daryl Granner and worked on the hexokinase-2 gene and its protein structure and function. Daryl Granner played an instrumental role in my life, teaching me the importance of fundamental and mechanistic science and approaching a scientific question from the right angle. After completing my MD and PhD, I moved to Baltimore and completed a residency in medicine and fellowship in cardiology. I did my fellowship research in the lab of Eduardo Marban, who was at the time the Chief of Cardiology at Hopkins and supported me in my research aspiration and my goal to become an independent investigator.

During graduate school, I got to know and marry my wife, and that was without a doubt the best event of my life. My wife, Fatemeh M. Ardehali, has been my best friend and companion for the past 27 years. My daughter, who is now a medical student at Duke, was born while I was in medical school, and my son, who is now a premedical student at Northwestern, was born while I was a resident at Johns Hopkins. They are both the joys of my life and I am proud of the great children my wife and I have raised together (Figure 3). They both experienced first-hand how much I enjoy my life and my career as a physician-scientist, which reinforced their decisions to pursue careers as physicians.

After completing my fellowship training, I chose to move to Northwestern University as an Assistant Professor in 2005. I moved up through the ranks and became a Professor of Medicine and Pharmacology in 2014 and Thomas D. Spies Professor in 2019. I am also the Director of the MSTP, a position I hold dearly since it allows me to be part of the training of the future physician-scientists. It is an extremely enjoyable experience to interact with the young students in our program, as I highly value training young physician-scientists and assisting them in their challenging paths to success.

I have benefited from the mentorship of a number of individuals throughout my career. In addition to my PhD and fellowship mentors (Daryl Granner and Eduardo Marban), there are other names worth mentioning: David Robertson (MSTP director at Vanderbilt), Robert Bonow and Larry Jameson (my Chief of Cardiology and Chair of Medicine when I moved to Northwestern), and my current colleagues and mentors Doug Vaughn, Susan Quaggin, Eric Neilson, Alfred George, and Beth McNally.

My life has gone through a number of peaks and troughs and has spanned many years of significant turmoil. I have seen the hopes of people of the country where I was born in peace and freedom destroyed by a bloody war and with plentiful instances of the naked exercise of brutal power. I have witnessed their faith in a civilized society crumbled by a totalitarian regime and its organized cruelty. This has been a major source of disappointment to me. However, I have also experienced the society and the culture of the United States, where every person is given the full privileges of a free human being and can learn about different creeds, discuss issues, and choose the principles that are most consistent with their beliefs. Unlike countries with totalitarian regimes, the US allows freedom of speech, religion, press, and belief. These personal experiences have had a profound impact on me and have encouraged me along the path of “the pursuit of knowledge.” These experiences and my desire to learn new findings solidified my goal to pursue a career in medicine and science. I do not believe that I would have been at my current position if it were not for the obstacles I have experienced in my life. My life experiences have taught me to be vigilant and not to take the opportunities I have in my life for granted.

## Impact of immigration on science and technology

It is important to emphasize that I am one of so many immigrants who chose to devote their careers to science and technology. With so much anti-immigration campaigning in the past few years, it is important to highlight how much immigration has actually benefited science in this country. Contrary to what a number of political leaders and news media have promoted that has led to a significant amount of misinformation on immigration, this country and its scientific achievements are, at least partially, dependent on its immigration policies.

Since 2000, immigrants have been awarded close to 40% of the Nobel Prizes won by American scientists in Chemistry, Medicine, and Physics (1). From 1901 to 2021, 35% of American scientists who won the Nobel Prize in Chemistry, Medicine and Physics have been immigrants (1). A study by the American Enterprise Institute and New American Economy demonstrated that for every 100 immigrants who earn their advanced degrees in science, technology, engineering, and mathematics (STEM), 262 new jobs are created in this country (2). The US also gives H-1B visas to foreign workers with specialty occupations. This mechanism has been criticized and scrutinized heavily in the past few years, but data support the benefit of this program. For every 100 H-1B skilled foreign workers, 183 jobs are created (2). Another study showed that increasing the number of H-1B visas by just 1% reduces unemployment rates in these professions by about 0.2% and increases wages (1). These data (along with more numbers that support these findings) demonstrate the importance of immigration to science and technology in this country. Science will more readily advance when we wisely open the doors of our open society to skilled and educated workers.

## New ASCI initiatives

My background and my immense interest in the success of ASCI and physician-scientists have prompted me to initiate several new programs since I took over the role of ASCI President:

### Closer interaction between ASCI Council and members

One of my goals was to improve our connection with ASCI members that goes beyond a once-a-year annual conference.

#### Quarterly newsletter.

The newsletter contains a message from the President along with once-a-year reports from the following: Diversity, Publishing, Physician-Scientist Development, Advocacy, and Development Committees. Additional items include summary of the budget (once a year), Council activities, recent national scientific/policy news, and ASCI office reports.

#### Monthly webinars.

**Figure 4:**
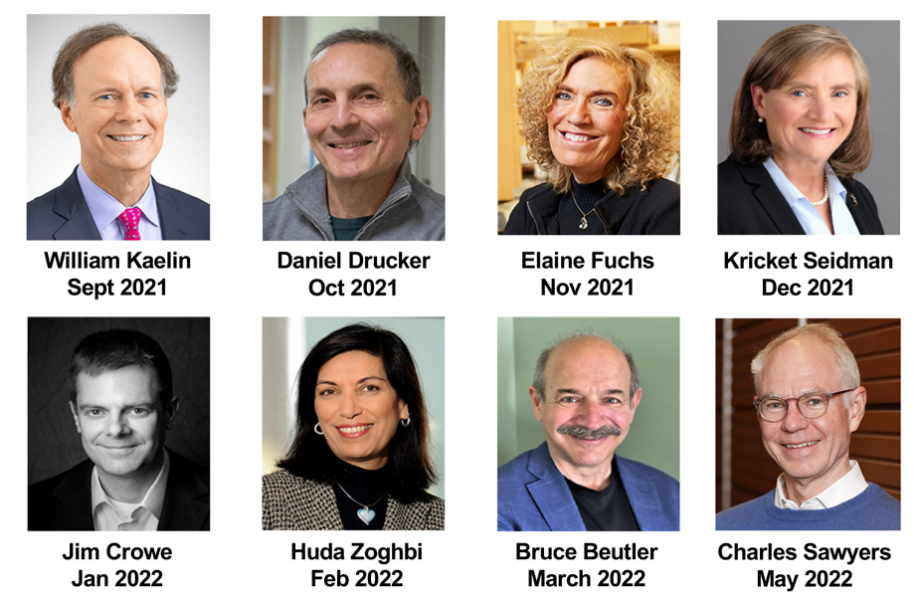
ASCI Scientific Sessions speakers for the 2021–2022 academic year.

Scientific Sessions are a monthly spotlight on distinguished investigators and their contributions to the field of biomedical research and are held eight times a year from September to May (Figure 4). They are free to everyone to attend and are not limited to ASCI members. Recorded sessions are also available on demand. 

### Expanding the number of new ASCI inductees and international and honorary members

We have recently had a number of highly qualified applicants who could not be inducted due to the limit of 80 new inductees. Upon recent approval by the ASCI membership, we increased the limit of new members to 100. This increase does not mean that the bar for ASCI membership is lowered; rather, the new limit provides the Council wider latitude to recognize a greater diversity of excellence in all areas where physician-scientists have an impact.

Unlike the National Academy of Sciences (NAS) and the National Academy of Medicine (NAM), ASCI membership is almost exclusively American physician-scientists. Thus, a key goal for me has been to create international collaborations. Building on work by past Presidents, including Mukesh Jain and Lorraine Ware, I have continued a conversation with Seamas Donnelly, the current President of the Association of Physicians of Great Britain and Ireland. The collaboration will likely lead to a number of new initiatives, including exchange of society presidents and a number of junior faculty/trainees to attend their respective annual conferences, establishment and expansion of international membership in both societies, establishment of an International Relationship Working Group, and a travel scholarship for scholars from both societies. We are hoping that this collaboration will continue to grow and will build the platform for collaboration with other international societies for physician-scientists.

### Improving career development programs for YPSA

Under the leadership of Anna Greka (Harvard Medical School) and Chris Williams (Vanderbilt), the Young Physician-Scientist Award (or YPSA) program has expanded tremendously in the past few years. This year, we introduced the Emerging Generation Awards (or E-Gen), which give prefaculty physician-scientists access to this meeting and additional longitudinal programming. This programming (for both YPSA and E-Gen) includes virtual poster sessions, career development workshops on networking and management, panel discussions on topics such as publishing and funding, and the new Physician-Scientist Pathway Series, where celebrated colleagues share their personal experiences on the physician-scientist career path with this group.

### Use of social media

ASCI has expanded its presence on Twitter, which we have adopted as our social platform. Our journals highlight major papers, and ASCI continues to make major announcements on Twitter.

### New awards

With seed funding provided by the ASCI Council, we are launching two new awards, with the first recipients honored in 2023: (a) The ASCI/Marian W. Ropes, MD, Award recognizes the significant scholarly achievements of a middle-career woman physician-scientist. The recipient of the annual award is provided with a $10,000 honorarium and gives the annual ASCI/Marian W. Ropes, MD, Award Lecture at the ASCI’s annual meeting; (b) the ASCI/Louis W. Sullivan, MD, Award recognizes the significant scholarly achievements of a middle-career physician-scientist who is underrepresented in medicine and science. The recipient of the annual award is provided with a $10,000 honorarium and gives the annual ASCI/Louis W. Sullivan, MD, Award Lecture at the ASCI’s annual meeting.

## COVID-19 and its impact on ASCI

It would be remiss of me to end this presidential address without discussing the impact of COVID-19 on our Society. Future generations of ASCI members will someday look at this speech/article and discuss the impact of COVID-19 from a historical perspective. COVID-19 has brought an unprecedented challenge to science and medicine in our country and around the world. As I am writing this note in the summer of 2022, more than 6.3 million people have died from the disease and 539 million people have been infected. We experienced the biggest surge of the Omicron variant, and many ICU rooms in the country were filled to their capacity in January of 2022.

Despite these challenges, there are some successes worth celebrating during this pandemic. The speed of vaccine generation and their relative effectiveness against the development of serious illness is a major achievement of the 21st century. The disease became a public health issue in the beginning of 2020, and by the end of that year, vaccines were available. Additionally, a number of new pharmacological agents have been developed to reduce symptoms and improve disease recovery. Development of monoclonal antibodies has also been a major step forward. Finally, the use of multi-omics has led to major advances in our understanding of the virus and its infection.

Despite the challenges this virus has brought to the whole world, there is another aspect of this pandemic that cannot be ignored, and that is misinformation. A number of people in positions of high power have taken the liberty of making nonscientific statements about the virus and have misled the public on the severity of the disease and its potential treatments. There has been an ongoing major antivaccine campaign, and many ICU patients are either unvaccinated or immune-compromised. In our ICU at Northwestern, at certain points, up to 80% of the patients were unvaccinated individuals.

In addition to misinformation about the pandemic, many of our politicians have also continued to spread misleading information about the basics of our democracy. As a result, we witnessed anarchy and violence in our capital. When I left Iran more than 35 years ago, I never thought I would see the violence we witnessed on January 6, 2021, or the spread of false and fake news we have seen in the past 5 to 6 years. The violence and anarchy, along with the misinformation I have recently seen, parallel those that led to years of instability in many other countries. Based on my past experience, I totally understand how important, and at the same time fragile, democracy can be.

This misinformation campaign and attack against public servants (who advise society on the disease) has to stop. As the honor society of physician-scientists, we have an obligation to prevent the spread of wrong information and to promote science and facts. We are witnessing the active spread of misinformation on “public health” and “political” issues, and I believe that ASCI members (as physicians, scientists, and educators) should use their voices to combat this practice that threatens public health and the basics of our democracy. Thus, I encourage everyone who is listening now, or who reads this article, to take an active role to protect our democracy and to support the promotion of science and facts.

## ASCI office staff

**Figure 5:**
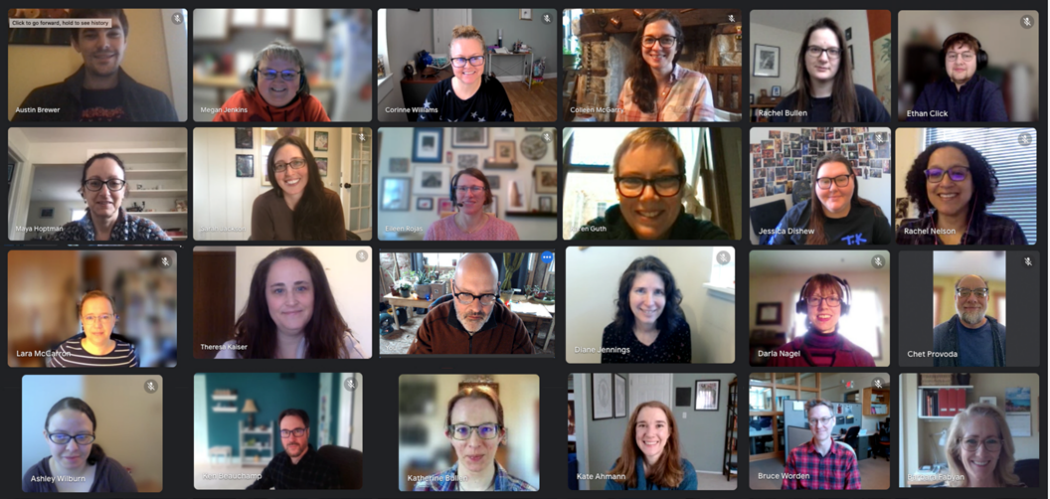
ASCI office staff at a recent Zoom meeting.

**Figure 6:**
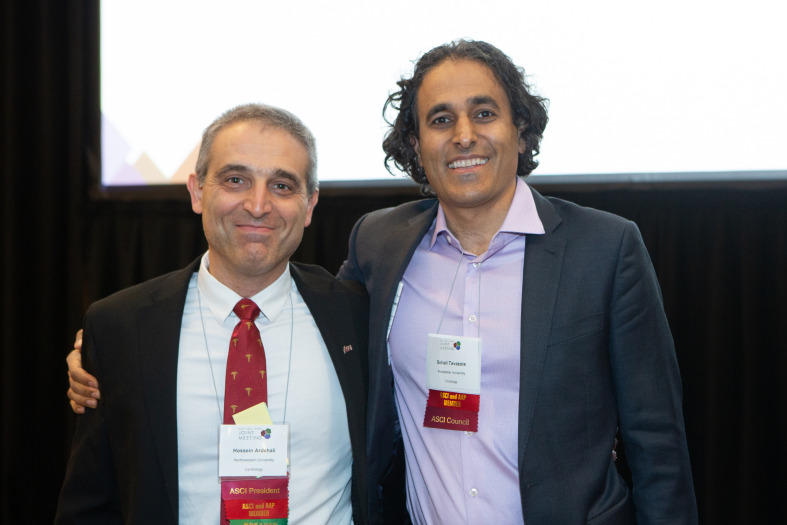
Incoming ASCI President, Sohail Tavazoie.

I strongly believe that one section of my presidential address should be devoted to the ASCI administrators and office staff. As detailed above, I introduced a number of major initiatives during my presidency years, but it was John Hawley, Karen Guth, and Colleen McGarry who built the foundation for these programs. A huge special thank you goes to my friend and colleague John Hawley, who never said no to my requests and always goes out of his way to improve ASCI. ASCI is very fortunate to have such a superb staff (Figure 5). Finally, I want to welcome the incoming ASCI president, my friend and colleague Sohail Tavazoie (Figure 6). ASCI will be in great hands under his leadership.

## Conclusions

When I was a second-year medical student at Vanderbilt, one of my pathology professors, the late Dr. Robert Collins, called on one of my classmates on the last day of class and asked him to tell the whole class why he was “lucky.” Dr. Collins gave the answer himself and told us that we were lucky for two reasons: (a) we are healthy, and (b) we live in this country. Having seen so many sick patients throughout my career, I totally agree with the first statement. As someone who has experienced another oppressive society, I also completely agree with the second point. I believe that I am the most fortunate individual for living in the US and to have the opportunity to conduct research along with patient care. I consider the US not only the greatest country in the world (despite its many shortcomings), but the most open and free society in the history of mankind. We should all consider ourselves “lucky” to do the work we do with the freedoms we possess. I certainly do consider myself lucky for all the privileges that I have received from the freedom I have experienced in this country.

## Figures and Tables

**Figure 1:**
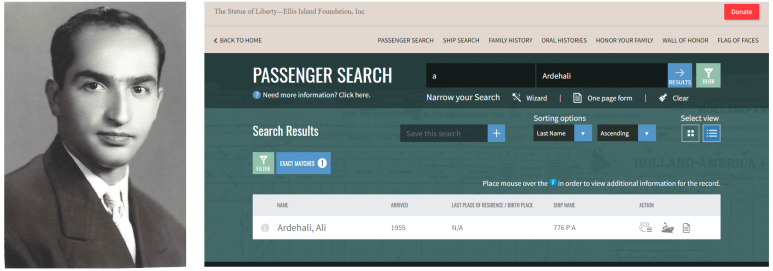
My father in his mid 20s when he immigrated to the US and his travel records from the Ellis Island Foundation. The year of his entry to the US was 1948, which is recorded incorrectly as 1955 in the image.

**Figure 2:**
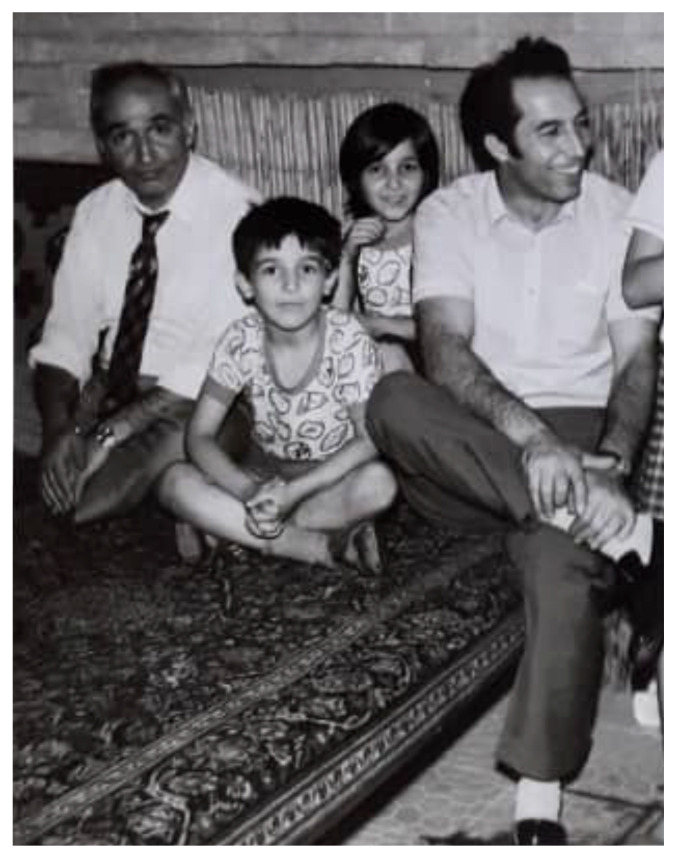
Picture of me along with my father, my uncle, and my sister the night before our older sister (not in the picture) immigrated to the US. I was 6 years old at the time.

**Figure 3:**
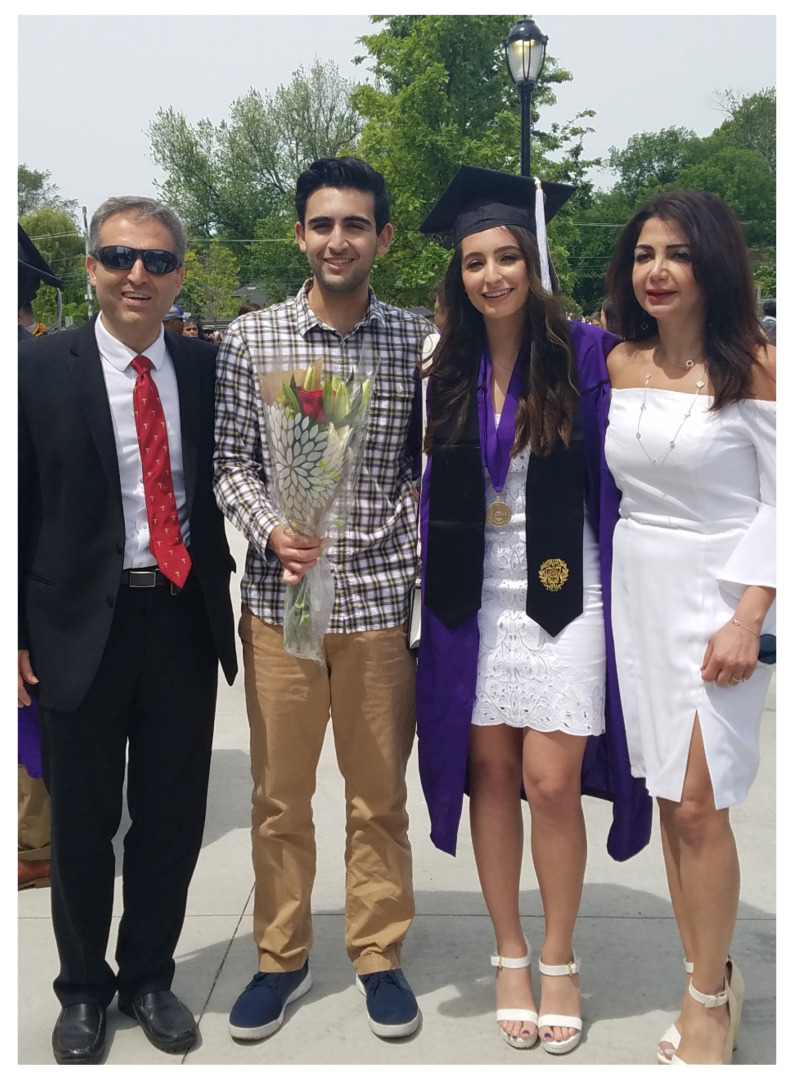
My family at my daughter’s graduation from Northwestern University. Spring 2019.

## Version 1 12/15/2022

Electronic publication
